# The effect of propolis on 5-fluorouracil-induced cardiac toxicity in rats

**DOI:** 10.1038/s41598-022-12735-y

**Published:** 2022-05-23

**Authors:** Mohammad Barary, Rezvan Hosseinzadeh, Sohrab Kazemi, Jackson J. Liang, Razieh Mansoori, Terence T. Sio, Mohammad Hosseini, Ali Akbar Moghadamnia

**Affiliations:** 1 Student Research Committee, Virtual School of Medical Education and Management, Shahid Beheshti University of Medical Sciences, Tehran, Iran; 2 Students’ Scientific Research Center (SSRC), Tehran University of Medical Sciences, Tehran, Iran; 3 Student Research Committee, Babol University of Medical Sciences, Babol, Iran; 4 Cellular and Molecular Biology Research Center, Health Research Center, Babol University of Medical Sciences, Babol, Iran; 5 Division of Cardiovascular Medicine, Cardiac Arrhythmia Service, University of Michigan, Ann Arbor, MI USA; 6 Department of Radiation Oncology, Mayo Clinic, Phoenix, AZ USA; 7 Department of Veterinary Parasitology, Babol-Branch, Islamic Azad University, Babol, Iran

**Keywords:** Cancer, Cardiology

## Abstract

5-Fluorouracil (5-FU) is one of the most common chemotherapeutic agents used in treating solid tumors, and the 5-FU-induced cardiotoxicity is the second cause of cardiotoxicity induced by chemotherapeutic drugs. Propolis (Pro) has vigorous anti-inflammatory activity. Its cardio-protective characteristic against doxorubicin-induced cardiotoxicity was previously proven. The current study aimed to appraise the effect of Pro on 5-FU-induced cardiotoxicity in rats. Twenty-four male Wistar rats were divided into four groups: Control, 5-FU, 5-FU + Pro 250 mg/kg, and 5-FU + Colchicine (CLC) 5 mg/kg. Different hematological, serological, biochemical, histopathological, and molecular assays were performed to assess the study’s aim. Moreover, a rat myocardium (H9C2(2–1)) cell line was also used to assess this protective effect in-vitro. 5-FU resulted in significant cardiotoxicity represented by an increase in malondialdehyde (MDA) levels, cyclooxygenase-2 (COX-2) and tumor necrosis factor-α (TNF-α) expression, cardiac enzyme levels, and histopathological degenerations. 5-FU treatment also decreased bodyweight, total anti-oxidant capacity (TAC), catalase (CAT) levels, blood cell counts, and hemoglobin (Hb) levels. In addition, 5-FU disrupted ECG parameters, including increased elevation in the ST-segment and increased QRS complex and QTc duration. Treating with Pro reduced oxidative stress, cardiac enzymes, histopathological degenerations, and COX-2 expression in cardiac tissue alleviated ECG disturbances and increased the number of blood cells and TAC levels. Moreover, 5-FU-induced bodyweight loss was ameliorated after treatment with Pro. Our results demonstrated that treatment with Pro significantly improved cardiotoxicity induced by 5-FU in rats.

## Introduction

Cardiovascular diseases (CVDs) are a significant cause of death globally and substantially burden countries. In 2015, about 18 million deaths worldwide were due to CVDs, half of which were caused by ischemic heart disease (IHD)^1^. It has been proven that atherosclerosis is one of the significant risk factors for coronary artery disease (CAD), and in turn, inflammation has also been proven to play a vital role in the manifestation of atherosclerosis^2^. Thus, several chemotherapeutic agents, by inducing inflammation and subsequent inflammatory response in the body, can cause severe cardiotoxicity, including myocardial ischemia^3^. Angiogenic inhibitors, such as bevacizumab^4^, sunitinib, and sorafenib^5^, and also direct ABL inhibitors, including imatinib^6^ and dasatinib^7^, can cause myocardial ischemia. Other essential and widely-used chemotherapeutic drugs with cardiotoxicity side effects are doxorubicin^8^ and 5-FU^9^.

5-fluorouracil (5-FU), a member of the fluoropyrimidine class of chemotherapeutic drugs, can cease DNA replication by inhibiting the formation of thymidine by several intracellular mechanisms, of which inhibition of thymidylate synthase enzyme seems to play the significant role^10^. Since its introduction in 1957 by Heidelberger et al.^11^, it has been an essential part of chemotherapy regimens for several solid tumors, including gastrointestinal, breast, head and neck, and pancreatic neoplasms^12^. It can cause many side effects, including severe cardiotoxicity, often manifested as myocardial ischemia, but can also be presented as cardiac arrhythmias, hyper- and hypotension, left ventricular dysfunction, cardiac arrest, and even death^13^. The prevalence of 5-FU-induced cardiac complications can vary between 0–20%, mainly depending on the dosage, comorbidity factors, and schedule of the chemotherapy regimen^9^. The definite mechanisms of 5-FU-induced cardiotoxicity are not clearly understood. However, some studies suggest that coronary artery thrombosis, arteritis, or vasospasm may be responsible for its adverse effects^13^. Other mechanisms have also been reported to be responsible for this drug’s cardiotoxicity, including direct myocardial toxicity^14^, activation of autoimmune responses^15^, and direct coronary endothelial intima toxicity^16^. Several known polyphenols have proven cardio-protective characteristics, including resveratrol^17^, quercetin^18^, catechins^19^, curcumin^20^, baicalein^21^, genistein^22^, and apigenin^23^. Also, propolis is another agent with proven cardioprotective effects^24^.

Propolis, also known as bee glue, is a lipophilic resinous agent that honey bees produce while constructing their hives. Briefly, bees collect different botanical materials, such as mucilage, gums, resins, and lattices. When these materials mix with the enzyme β-glycosidase secreted in bees’ saliva, they undergo partial digestion, and when added to beeswax, they eventually form raw propolis known as propolis in nature^25^. The nature of this final product alters in different temperatures so that it becomes hard and wax-like when cool but soft and sticky when warm. Components of Propolis, and as a result, its biological activities, can vary widely based on where it is made and the plant variability around the hive^25^. It can be made mainly of phenolics, like flavonoids, aromatic acids, and their ester in temperate regions, to prenylated *p*-coumaric acid derivatives in Brazilian propolis^25^. Some of its proven biological activities include: anti-inflammatory, anti-oxidant, anti-ulcerogenic, hepatoprotective^26^, antitumor^27^, immune-stimulation/modulation^28^, antibacterial^29^, antiviral^30^, antifungal^31^, and anti-parasite^32^. Its cardio-protective characteristic against doxorubicin-^24^, hypothermic-^33^, N $\omega$-nitro-L-arginine methyl ester (L-NAME)-induced cardiotoxicity^34^ has also been previously investigated.

This study investigates the ameliorative effects of propolis administration in 5-fluorouracil-induced cardiotoxicity in-vitro and in-vivo.

## Materials and methods

### Chemicals

All chemicals were of the highest grade (analytical grade). N-hexane (HPLC grade), ethanol (purity > 98%), N, O-Bis(trimethylsilyl)trifluoroacetamide (BSTFA) with 1% trimethylsilyl chloride (TMCS), polyethylene glycol (M_n_: 6000), and castor oil were purchased from Merck KGaA, Darmstadt, Germany. Dimethyl sulfoxide (DMSO, GC grade, purity > 99.99%) was acquired from Sigma-Aldrich Co., St. Louis, MO, USA. 5-fluorouracil 1000 mg vial was purchased from Iran Darou, Tehran, Iran. Ultrapure Milli-Q water (18.2 MW) was used in all experiments.

### Propolis origin and collection

*Apis mellifera* bee hives’ crude propolis were collected in Spring 2020 in the Alborz mountains in Polur, Tehran, Iran. All propolis samples were kept at 4 °C, protected from light until further extract preparation.

### Propolis extraction

The extraction method was based on a previous study^35^. The propolis samples were powdered by a mortar and pestle, and the powdered samples were mixed with n-hexane at the ratio of 3:100 (w/v; 3 g of crude propolis was mixed with 100 mL of n-hexane) and shook (120 rpm) at 30 °C for 4 days to remove the bee wax. The mixture was filtered by Whatman 42 filter paper (Sigma-Aldrich Co., St. Louis, MO, USA), and the remaining solid parts of propolis samples on the filter paper were dried at room temperature. After removing bee wax, the solid residues were extracted with two different solvents, 70% ethanol (EtOH) and DCM, with a ratio of 3:10 (w/v). After hexane extraction, a 3 g sample was dissolved in 10 mL of 70% EtOH, and the extraction was carried out in the dark on a shaker (120 rpm) at 30 °C for 3 days. The 70% EtOH extract of propolis was filtered by Whatman 42 filter paper under a vacuum. The organic solvent of the filtered extract was removed under reduced pressure at 50 °C by a rotary evaporator. The final extracts were stored in a sealed container in a refrigerator at 4 °C and protected from light until gas chromatography-mass spectrometry (GC–MS) analysis^36^.

### Derivatization procedure

Five grams of the final Propolis ethanolic extract were dissolved in 250 µL of pyridine (anhydrous, purity = 99.8%) and 500 µL of BSTFA, including 1% TMCS, and shook at 100 °C for half an hour.

### Gas chromatography-mass spectrometry (GC–MS) analysis of propolis ethanolic extract

The detailed information on this extract’s botanical origin and phytochemical constituents are discussed elsewhere^37^. Nevertheless, the chemical composition of Propolis ethanolic extract was assessed again using a gas chromatography–mass spectrometry device (5977B GC/MSD, Agilent, Santa Clara, CA, USA). The DB-5 ms capillary column experimental conditions were as follows: length = 30 m, inner diameter = 0.25 mm, film thickness = 0.25 µm, gas carrier: Helium with purity ≥ 99.9995% (Sigma-Aldrich Co., St. Louis, MO, USA), gas flow rate: 1 mL/min. The GC–MS analysis was based on a previous study^37^. Briefly, 1 µL of the final solution prepared in the last step was injected into the device with an autosampler in a split 10:1 ratio. The temperature of the injector was set at 250 °C. The oven temperature was programmed to start from 50 °C (storage time of 1 min), then increase 8 °C/min rate up to 120 °C (storage time of 1 min). Finally, the temperature was to increment at a 6 °C/min rate up to 250 °C in about 15 min. The total device running time was 47 min, with a solvent delay of 0–3 min. The propolis sample components’ names, molecular weight, and structure were identified using the National Institute of Standards and Technology (NIST 11 Variant) database.

### Cell cultures

Rat myocardium (H9C2(2–1)) cell line was obtained from the National Cell Bank of Iran (NCBI, Pasteur Institute, Tehran, Iran). These cell lines were cultured in Roswell Park Memorial Institute medium (RPMI 1640) containing glucose 2 g/L, L-arginine, and L-glutamine 200 and 300 mg/L, respectively, supplemented with 10% fetal bovine serum (FBS) and 1% pen/strep comprising of penicillin G 100 U/mL, and streptomycin 100 µg/mL. The cell lines were grown as monolayers in 25 cm^2^ cell culture flasks at 37 °C in a 5% CO_2_ humidified atmosphere.

### Cell viability assay

The cytotoxicity of Propolis ethanolic extract was assessed with the cell viability (MTT) assay. The method for MTT assay used in this study was described elsewhere^38^. Briefly, the cell line was treated with 5-FU 75 µM, CLC 50 µg/mL, and Pro 50, 100, and 200 µg/mL, with each concentration investigated in triplicates. The treated plates were then incubated at 37 °C in a 5% CO_2_ humidified atmosphere, and after 48 h, an MTT assay was performed. Then, a 5 mg/mL concentration of MTT dye (Alfa Aesar, Thermo Fisher (Kandel) GmbH, Kandel, Germany) was mixed with phosphate-buffered saline (PBS), the mixture was filtered by Whatman grade 42 ashless quantitative filter papers (Sigma-Aldrich Co., St. Louis, MO, USA), and 50 µL of the filtered solution was added to each seeded well and incubated at 37 °C for 4 h. After removing the supernatant, 150 µL of DMSO was added to each well, and the optical density (OD) was measured by an absorbance microplate reader (ELx808, BioTek, Winooski, VT, United States) at 570 nm.

### Animal experimental design

Twenty-four male Wistar rats (6–8 weeks, 180 ± 20 g) were obtained from Babol University of Medical Sciences’ laboratory animal facility. All animals were kept in 12-h light/12-h dark cycles and housed in a controlled environment with specified temperatures (22–24 °C) and humidity (50 ± 5%). Animals were kept in cages with LSB Aspen woodchip bedding and had free access to food and tap water during the whole experimental period. All study procedures were conducted following the approval of the National Institute for Medical Research Development (NIMAD) ethical board (Code: IR.NIMAD.REC.1399.255).

Study rats were randomly divided into four groups as follows:

- The negative Control group received 200 µL of castor oil for 14 days (Control).
- The 5-fluorouracil group received a bolus 125 mg/kg dosage of 5-fluorouracil intraperitoneally (5-FU).
- The experimental group received a bolus 125 mg/kg dosage of 5-fluorouracil intraperitoneally plus propolis ethanolic extract 250 mg/kg/d by oral gavage for 14 days (5-FU + Pro).
- The positive control group received a bolus 125 mg/kg dosage of 5-fluorouracil intraperitoneally plus colchicine 5 mg/kg/d by oral gavage for 14 days (5-FU + CLC).

### The electrocardiography (ECG)

Electrocardiography (ECG) was performed for 15 min the day before euthanizing the study animals. For this purpose, the rats in each group were anesthetized with ketamine/xylazine, subcutaneous peripheral limb electrodes were inserted into the limbs to record the standard lead II of the electrocardiograph, and ECG parameters, such as ST-segment elevation and QRS and QTc duration, were measured using an ECG device (eLab, Sciencebeam, Tehran, Iran)^39^.

### Sample collection and preparation

Rats were weighed and anesthetized with ketamine/xylazine on the fifteenth day. Then, 5 mL blood samples were immediately collected directly from the heart and poured into 5 mL microtubes for further serum separation by centrifugation for 15 min at 1,500 g. Afterward, the animals were euthanized by the decapitation method, and their heart was harvested. About 20- 30 mg of the harvested heart tissue were immediately transferred to 1.5 mL RNase and DNase-free microtubes, including 200 µL RNA later solution (Yekta Tajhiz Azma, Tehran, Iran). After overnight incubation at 4 °C, these microtubes were transferred to − 80 °C until the RNA extraction. However, the remaining tissue samples were placed in a formalin-containing tube and then, along with serum samples, were kept at − 20 °C for further analysis.

### Laboratory analysis

#### Complete blood count (CBC)

The white blood cells (WBC, × 10^3^/µL), and red blood cells (RBC, × 10^6^/µL) count, hemoglobin (Hb, g/dL), and platelets (PLT, × 10^3^/µL) were quantified with an automated counter (H9000, Xuzhou forward medical instrument Co. Ltd., Xuzhou, China).

#### Serological analysis

As to determine the enzymatic activity of liver tissue, the levels of liver function tests (LFT), i.e., aspartate aminotransferase (AST) and alanine aminotransferase (ALT), were evaluated by commercial ELISA kits (Pars Azmun, Karaj, Iran). Then, De Ritis (AST/ALT) ratio was calculated for the study samples.

#### Cardiac marker enzymes assay

Lactate dehydrogenase (LDH, IU/L) and creatinine kinase-MB (CK-MB, IU/L) activities were measured in serum samples by commercial ELISA kits (Pars Azmun, Karaj, Iran).

### Biochemical analysis

#### Total anti-oxidant capacity (TAC) assay

A commercial enzyme-linked immunosorbent assay (ELISA) kit (Teb Pazhouhan Razi, Tehran, Iran) was used to assess the total anti-oxidant capacity (TAC) of the rats’ serum samples. Miller et al. (1993) published a detailed description of the technique^40^. Finally, an absorbance microplate reader (ELx808, BioTek, Winooski, VT, United States) measured the OD of the samples at 420 nm. This ELISA kit’s intra- and inter-assay coefficient of variation was 5.7% and 3.7%, respectively, and its detection range was 45–420 μM.

#### Catalase (CAT) assay

Catalase (CAT) is a ubiquitous anti-oxidant enzyme present in all cells’ peroxisomes, providing cell protection against oxidative stress-induced damage by catalyzing the decomposition of hydrogen peroxide (H_2_O_2_) to water and oxygen. A commercial ELISA kit was used to assess CAT levels (Teb Pazhouhan Razi, Tehran, Iran) in which CAT activity was assessed by the reaction of the CAT present in the sample with methanol in the presence of an optimal concentration of H_2_O_2_ to produce formaldehyde. After adding a chromogen that turns aldehydes purple, formaldehyde formation is determined by colorimetric analysis. Finally, an absorbance microplate reader (ELx808, BioTek, Winooski, VT, United States) measured the OD of the samples at 540 nm. This kit’s intra- and inter-assay coefficient of variation was 4.1% and 9.9%, respectively.

#### Malondialdehyde (MDA) assay

Malondialdehyde (MDA) assay was used to evaluate the lipid peroxidation levels of the serum samples. MDA is an end product of the oxidative decomposition of the polyunsaturated fatty acids initiated by free radicals. Thus, it is a frequently measured biomarker of oxidative stress. A commercial ELISA kit was used to assess MDA levels (Teb Pazhouhan Razi, Tehran, Iran) using a spectrophotometric method based on the reaction between MDA and thiobarbituric acid (TBA), generating an MDA-TBA adduct, which can be quantified by colorimetric analysis. Finally, an absorbance microplate reader (ELx808, BioTek, Winooski, VT, United States) measured the OD of the samples at 540 nm. This ELISA kit’s intra- and inter-assay coefficient of variation was 6.7% and 7.2%, respectively, and its detection range was 0–50 μM.

### Histopathological analysis

Each rat’s heart was harvested and weighed separately. These tissue specimens were fixed in 10% formalin solution and processed using a tissue processing device (dewatering, clearing, and staining), embedded in paraffin blocks, sliced in 5 µm thicknesses layers, and stained with hematoxylin and eosin (H&E). An average of four sections was placed on each slice. Therefore, approximately 390 sections were evaluated with digital light microscopy. Hyperemia, necrosis, and hyalinization were assessed in each section. An experienced user (Seyed Mohammad Hosseini) performed all morphological analyses using a Medicus pro-Myko microscope (Helmut Hund GmbH, Wetzlar, Germany) under × 40, × 100, and × 400 magnifications.

### RNA extraction

A total RNA extraction commercial kit (Pars Tous Biotechnology, Mashhad, Iran) extracted the total RNA of the previously described 20–30 mg of harvested heart tissue. The extracted RNA of each sample was measured using a NanoDrop spectrophotometer (Thermo Scientific, Waltham, MA, USA). Then, all RNA samples were transferred to − 80 °C until further analysis.

### cDNA synthesis

For cDNA synthesis, a commercial cDNA synthesis kit (Pars Tous Biotechnology, Mashhad, Iran) was used in which the following mixture was included: 250 ng of the previously mentioned extracted RNA samples, 5 µL of the 2 × enzyme buffer, and 1 µL of the reverse transcriptase enzyme. The resulting mixture then reached a 10 µL volume using diethylpyrocarbonate (DEPC)-treated water. Afterward, the mixture was incubated with a PCR-thermocycler (FlexCycler^2^, Analytik Jena AG, Jena, Germany) as follows: At room temperature for 10 min for the random hexamer primer annealing, at 47 °C for 60 min for the reverse transcriptase reaction, and finally, at 85 °C for 5 min for the ending the reaction.

### Quantitative real-time PCR

cDNA samples were amplified in duplicates by PCR in RealQ Plus Master Mix Green (Ampliqon, Odense C, Denmark) using a 7300 Real-Time PCR System (Applied Biosystems, Thermo Fisher Scientific, Waltham, MA, USA). OLIGO Primer Analysis Software 7 (DBA Oligo, Inc., Colorado Springs, CO, USA) was used to design specific primers summarized in Table 1. Briefly, real-time PCR was performed using 10 µL of PCR reaction mixture consisting of 6.25 µL of master mix, 0.25 µL of each primer, 2.25 µL of RNase free dH_2_O, and 1 µL of cDNA templates. The amplification reaction cycles were performed as follows: Initial denaturation at 95 °C for 15 min, then 40 cycles at 95 °C for 15 s, annealing temperature for 30 s which was different for each primer as depicted in Table 1, and extension at 72 °C for 30 s. At the end of the amplification cycles, the temperature of the samples was increased at a steady rate of 0.2 °C/min from 60 °C to 95 °C for calculating the melting curve. Melting curve analyses and negative controls were embedded in each assay to ensure that the reaction contamination was not producing anyCR products^41^. The target genes’ relative expression ratios (R) were measured using a model proposed by Pfaffl et al.^42^, in which the reference and target genes’ efficiency was calculated according to a relative standard curve comprised of various dilutions (i.e., 1:1, 1:2, 1:4, 1:8, 1:16, and 1:32) of cDNAs from high-quality samples with good target genes expression. In this study, glyceraldehyde 3-phosphate dehydrogenase (GAPDH) was used as the reference gene to normalize samples.

**Table 1:** Primer sequences of *COX-2*, *TNF-α*, and the housekeeping genes.

Primer	5’-3’
COX-2 forward	CAACCAGCAGTTCCAGTATCAGA
COX-2 reverse	CAAGGAGGATGGAGTTGTTGTAGAG
TNF-α forward	AAATGGGCTCCCTCTCATCAGTTC
TNF-α reverse	TCTGCTTGGTGGTTTGCTACGAC
GAPDH forward	CTACATGGCCTCCAAGGAGTAAG
GAPDH reverse	CCTCCTCTTCTTCGTCTATGGC

*COX-2* Cyclooxygenase-2, *TNF-α* Tumor necrosis factor-α, *GAPDH* Glyceraldehyde 3-phosphate dehydrogenase.

### Statistical analysis

All data were presented as mean ± standard deviation (SD). One-way ANOVA followed by post hoc Tukey tests were used to assess differences between study groups. Moreover, for non-parametric analyses, such as histopathological evaluations, the Kruskal–Wallis, and Mann–Whitney U tests were utilized. A probability level (p-value) of < 0.05 were considered statistically significant. Nevertheless, wherever applicable, other p-values are presented, i.e., p < 0.01, < 0.001, and < 0.0001. All statistical analyses were performed with the SPSS v. 26 software (IBM Inc., Chicago, IL, USA).

### Ethics approval and consent to participate

The animal used in the following investigation was handled strictly according to the Animal (Scientific Procedures) Act 1986. Moreover, all study procedures were conducted following the approval of the National Institute for Medical Research Development (NIMAD) ethical committee (Code: IR.NIMAD.REC.1399.255). Moreover, this study is reported in accordance with ARRIVE guidelines (https://arriveguidelines.org/).

## Results

### The Gas chromatography-mass spectrometry (GC–MS)

**Figure 1:**
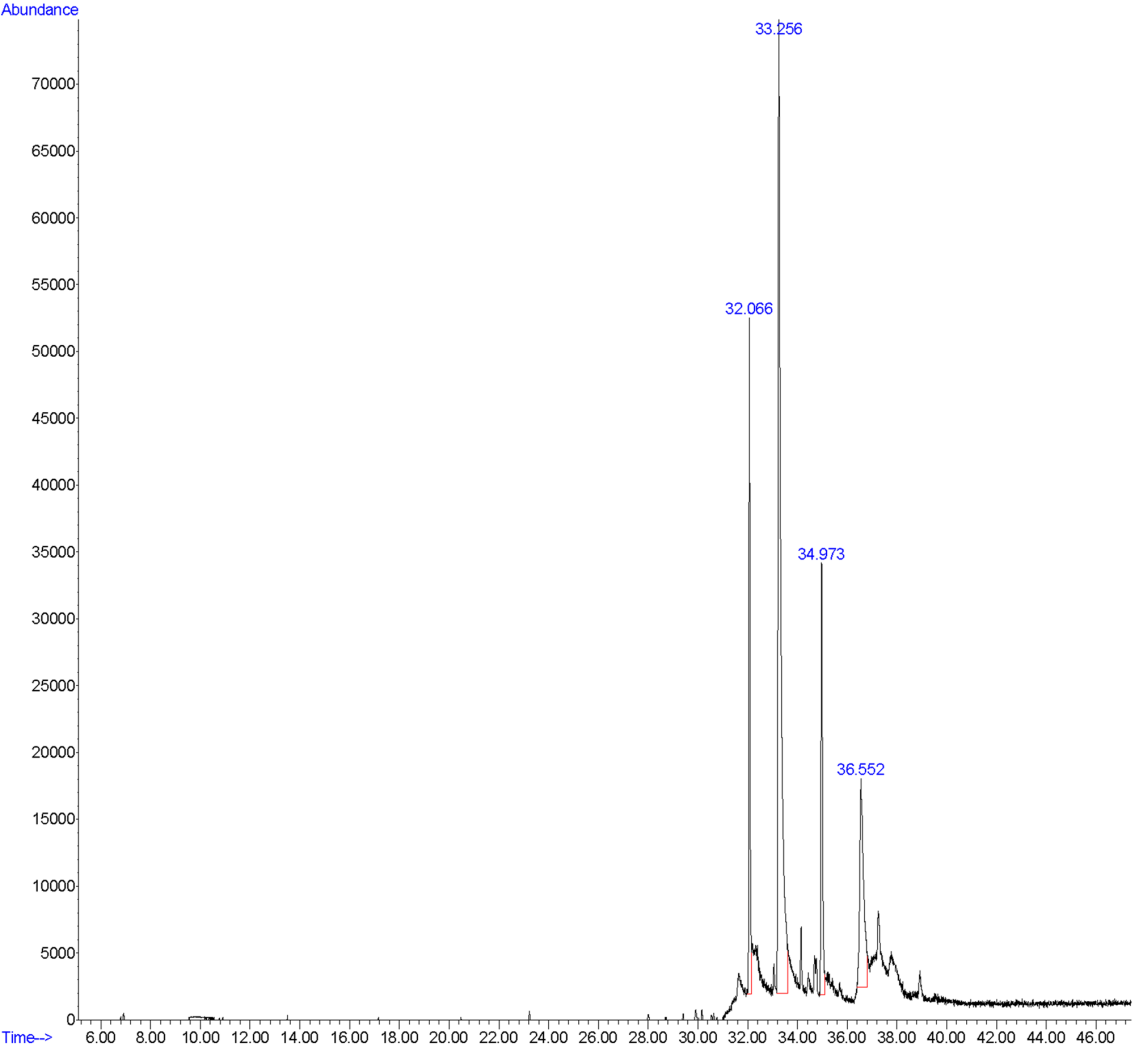
The GC–MS analysis of the propolis ethanolic extract. Sample peaks were detected as pinostrobin chalcone (32.066 s, 15.099% of total), galangin (33.256 s, 55.509% of total), tectochrysin (34.973 s, 13.216% of total), and naringenin (36.552 s, 16.177% of total).

In the GC–MS study, the first peak detected at 32.066 s was related to the pinostrobin chalcone (15.099% of total). The second, third, and fourth peaks were related to the galangin (55.509% of total, 33.256 s), tectochrysin (13.216% of total, 34.973 s), and naringenin (16.177% of total, 36.552 s), respectively (Fig. 1).

### The cell viability (MTT) assay

**Figure 2:**
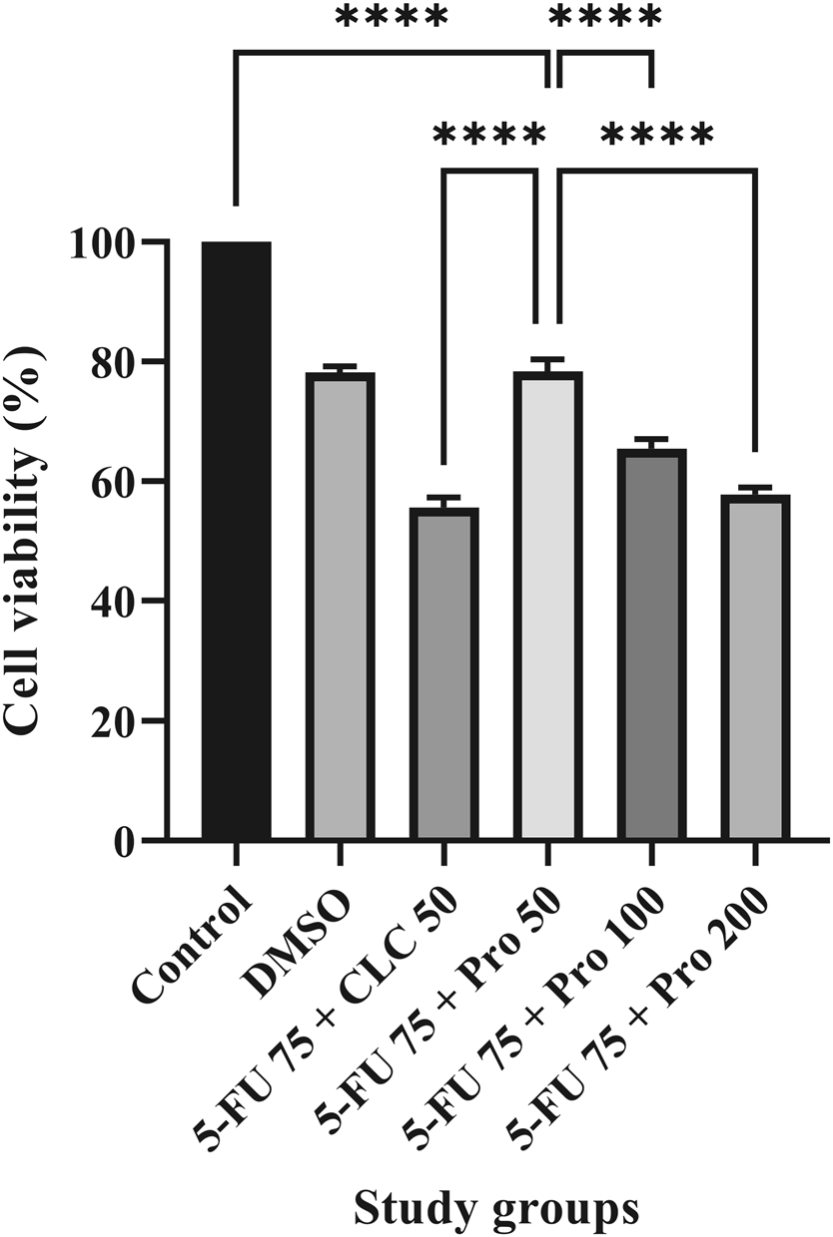
Effect of different doses of propolis on the viability of rat myocardium (H9C2(2–1)) cell line. All values are expressed in mean ± SD. ^****^ indicate p < 0.0001.

In the H9C2(2–1) cell lines, the highest and lowest levels of MTT were detected in the Control and 5-FU 75 + CLC 50 groups, respectively. The levels of the MTT were almost the same in DMSO and 5-FU 75 + Pro 50 groups. The cell survivability in 5-FU 75 + CLC 50, 5-FU 75 + Pro 100 and 5-FU 75 + Pro 200 groups were significantly lower than the 5-FU 75 + Pro 50 group (p < 0.0001). Also, the MTT level in the Control group was significantly higher than the 5-FU 75 + Pro 50 group (p < 0.0001). Moreover, it was observed that increasing concentrations of Pro would decrease the survivability of the cells in a dose-dependent manner, supposedly due to a synergistic toxic effect with 5-FU (Fig. 2).

### Relative heart weight of rats

**Figure 3:**
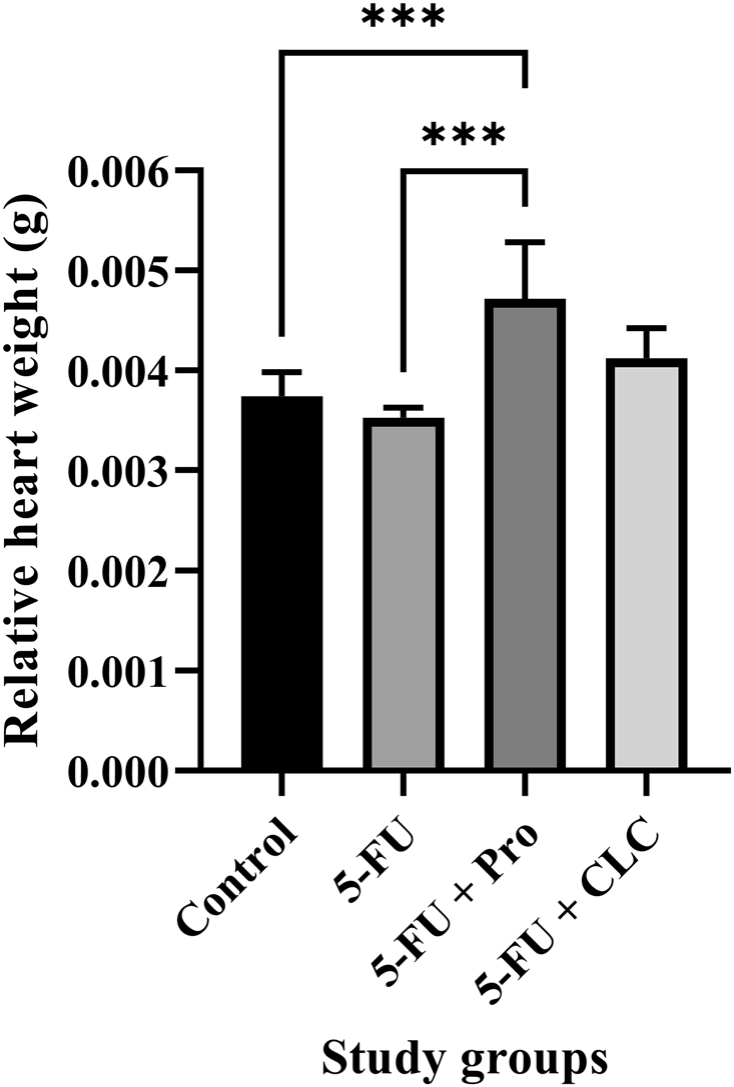
Effect of 5-flurouracil and propolis administration on the relative heart weight of rats. Values are expressed as mean ± standard deviation (SD). Groups: Control, Normal saline; 5-FU, 5-FU 125 mg/kg; 5-FU + Pro, 5-FU 125 mg/kg + Propolis ethanolic extract 250 mg/kg/d; 5-FU + CLC, 5-FU 125 mg/kg + Colchicine 5 mg/kg/d. ^***^ indicate p < 0.001.

The highest and lowest values of relative heart weight were observed in the 5-FU + Pro and Control groups, respectively. The relative heart weight of study rats was significantly higher in the 5-FU + Pro group than in the 5-FU and Control groups (p < 0.001). Moreover, the relative heart weight in the 5-FU and Control groups was lower than in the 5-FU + CLC group, but these differences were insignificant. The relative heart weight of rats in the 5-FU + Pro group was higher than the 5-FU + CLC group, but this difference was also not significant (Fig. 3).

### The electrocardiography parameters

#### QRS interval

**Figure 4:**
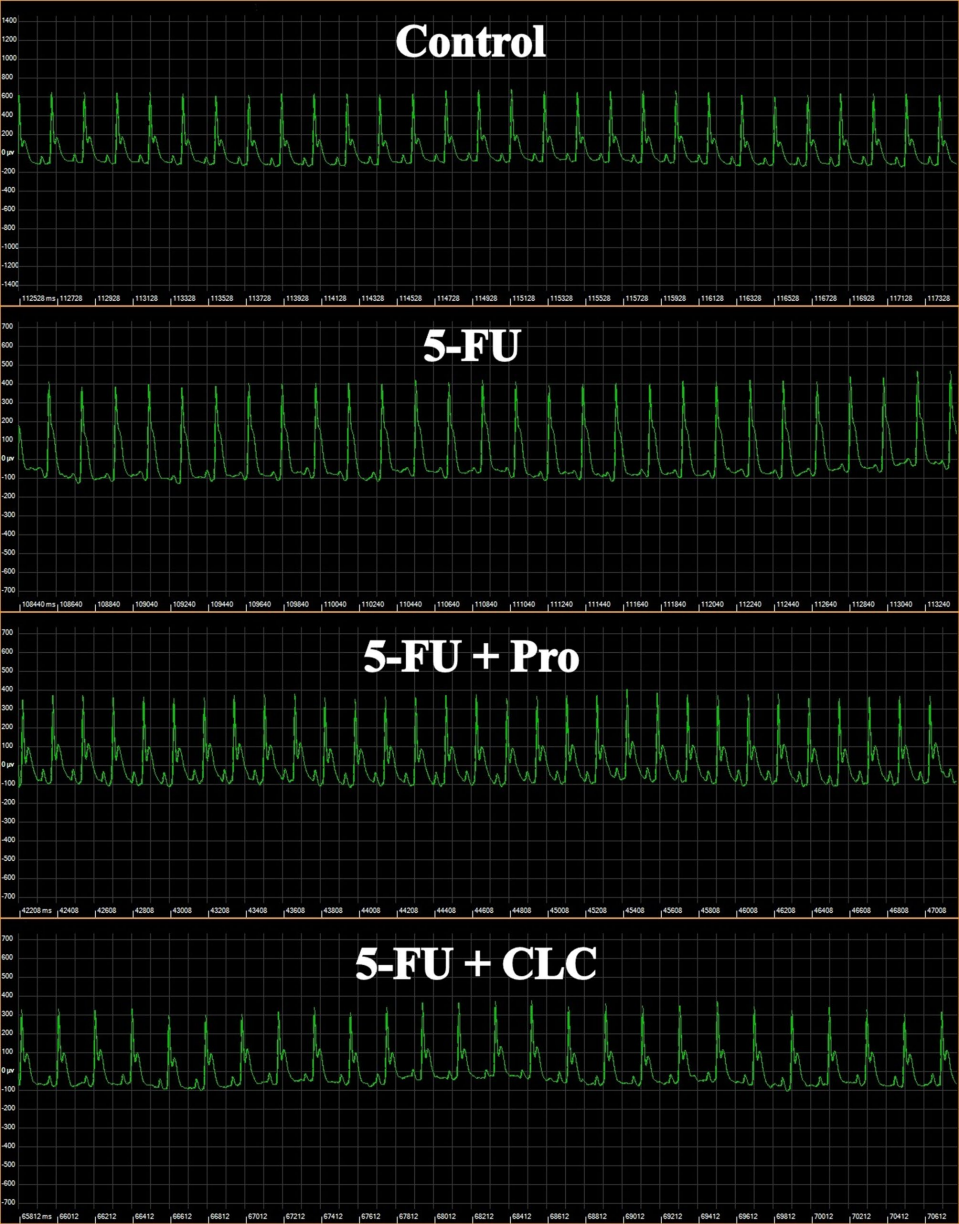
ECG images of study rats. Groups: Control, Normal saline; 5-FU, 5-FU 125 mg/kg; 5-FU + Pro, 5-FU 125 mg/kg + Propolis ethanolic extract 250 mg/kg/d; 5-FU + CLC, 5-FU 125 mg/kg + Colchicine 5 mg/kg/d.

The highest and lowest QRS intervals in the ECG examinations were measured in the 5-FU and 5-FU + Pro groups, respectively (Table 2). The QRS interval of the 5-FU and 5-FU + Pro groups was significantly higher than the Control group (p < 0.0001). Also, the QRS interval of the 5-FU + Pro and 5-FU + CLC groups was significantly lower than the 5-FU group (p < 0.0001). Moreover, the 5-FU + Pro group had a shorter QRS interval than the 5-FU + CLC group (p < 0.0001) (Fig. 4).

**Table 2:** Electrocardiogram parameters of study groups.

Study groups	ECG parameters		
	QRS interval (ms)	ST-segment (mV)	QTc (ms)
Control	12.38 ± 0.07	250.00 ± 22.21	115.60 ± 1.82
5-FU	14.82 ± 0.20****	457.80 ± 3.44****	142.60 ± 1.95****
5-FU + Pro	11.41 ± 0.18****^, ####, $$$$^	217.20 ± 9.11**^, ####, $^	114.20 ± 3.02^####, $$$^
5-FU + CLC	12.11 ± 0.20^####, $$$$^	241.70 ± 5.87^####, $^	106.80 ± 3.11****^, ####, $$$^

Values are expressed as mean ± standard deviation (SD). Groups: Control, Normal saline; 5-FU, 5-FU 125 mg/kg; 5-FU + Pro, 5-FU 125 mg/kg + Propolis ethanolic extract 250 mg/kg/d; 5-FU + CLC, 5-FU 125 mg/kg + Colchicine 5 mg/kg/d. **, and **** Indicate statistically significant difference compared to the control group (p < 0.01, and p < 0.0001, respectively). ^####^ Indicate statistically significant difference compared to the 5-FU group (p < 0.0001). ^$^, ^$$$^, and ^$$$$^ Indicate statistically significant difference between the treatment groups (p < 0.05, p < 0.001, and p < 0.0001, respectively).

#### ST-segment

In the ECG examinations, the highest and lowest ST-segment was detected in the 5-FU and 5-FU + Pro groups, respectively (Table 2). The 5-FU and 5-FU + Pro groups’ ST-segment was significantly higher than the Control group (p < 0.0001 and p < 0.01, respectively). Also, the 5-FU + Pro and 5-FU + CLC groups’ ST-segment were significantly less than the 5-FU group (p < 0.0001). Moreover, the ST-segment in the 5-FU + Pro group was significantly lower than the 5-FU + CLC group (p < 0.05) (Fig. 4).

#### QTc

It was observed that the highest and lowest QTc belonged to the 5-FU and 5-FU + CLC groups, respectively (Table 2). The QTc of 5-FU and 5-FU + CLC groups were significantly higher and lower than the Control group, respectively (p < 0.0001). Also, the QTc of 5-FU + Pro and 5-FU + CLC groups were significantly lower than the 5-FU group (p < 0.0001). Moreover, the QTc was significantly higher in the 5-FU + Pro group than in the 5-FU + CLC group (p < 0.001) (Fig. 4).

### The hematological parameters

#### Complete blood count (CBC)

##### White blood cell (WBC)

The highest and lowest WBC counts belonged to the 5-FU + Pro and 5-FU groups, respectively. WBC was significantly higher in the 5-FU + Pro group than in the 5-FU group (p < 0.05) (Table 3).

**Table 3:** Hematological factors of study groups.

Study groups	Hematological factors						
	WBC, × 10^3^/µL	RBC, × 10^6^/µL	Hb, g/dL	Plt, × 10^3^/µL	AST/ALT ratio	LDH, IU/L	CKMB, IU/L
Control	9.75 ± 2.24	7.10 ± 0.83	13.97 ± 1.14	643.83 ± 202.03	1.74 ± 0.42	1853.17 ± 728.11	68.67 ± 30.79
5-FU	8.16 ± 3.49	4.27 ± 0.59***	9.98 ± 2.43**	628.60 ± 258.47	3.71 ± 0.31****	2541.40 ± 544.56	102.60 ± 14.99
5-FU + Pro	22.48 ± 14.05^#^	5.24 ± 0.58**	11.10 ± 0.91*	1353.83 ± 249.26***^, ###^	2.89 ± 0.52**^, #, $^	2754.50 ± 771.68	105.50 ± 61.26
5-FU + CLC	11.53 ± 3.93	6.22 ± 1.26^#^	10.63 ± 0.77*	932.50 ± 297.09	2.13 ± 0.48^###, $^	1780.80 ± 460.55	53.00 ± 12.02

Values are expressed as mean ± standard deviation (SD). Groups: Control, Normal saline; 5-FU, 5-FU 125 mg/kg; 5-FU + Pro, 5-FU 125 mg/kg + Propolis ethanolic extract 250 mg/kg/d; 5-FU + CLC, 5-FU 125 mg/kg + Colchicine 5 mg/kg/d. *, **, ***, and **** Indicate statistically significant difference compared to the control group (p < 0.05, p < 0.01, p < 0.001, and p < 0.0001, respectively). ^#^, and ^###^ Indicate statistically significant difference compared to the 5-FU group (p < 0.05, and p < 0.001, respectively). ^$^ Indicate statistically significant difference between the treatment groups (p < 0.05).

##### Red blood cell (RBC)

The highest and lowest RBC counts were observed in the Control and 5-FU groups, respectively. The 5-FU + Pro and 5-FU groups’ RBC counts were significantly lower than the Control group (p < 0.01 and p < 0.001). Also, the RBC count of the 5-FU + CLC group was significantly higher than the 5-FU group (p < 0.05) (Table 3).

##### Hemoglobin (Hb)

The highest and lowest Hb levels were observed in the Control and 5-FU groups, respectively. The Hb level of the 5-FU + CLC, 5-FU + Pro, and 5-FU groups were significantly lower than the Control group (p < 0.05, p < 0.05, and p < 0.01, respectively) (Table 3).

##### Platelet (Plt)

The highest and lowest Plt counts were observed in the 5-FU + Pro and 5-FU groups, respectively. The Plt counts of the Control and 5-FU groups were significantly lower than the 5-FU + Pro group (p < 0.001) (Table 3).

#### Serological analysis

##### AST/ALT ratio

**Figure 5:**
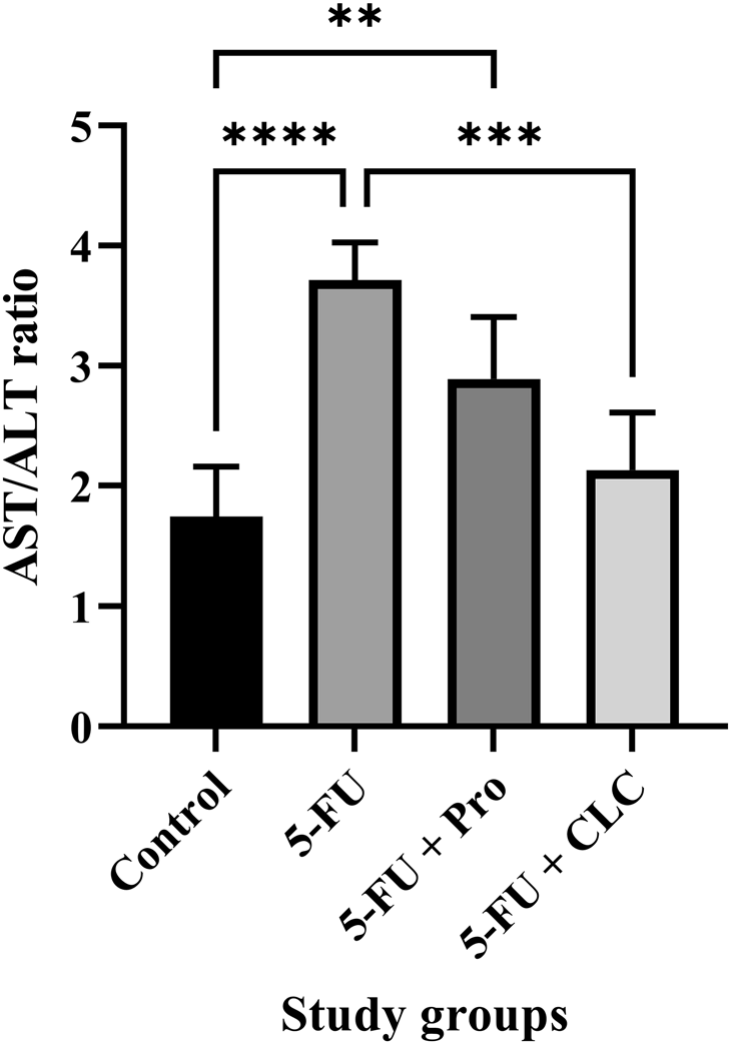
Effect of 5-flurouracil and propolis administration on the AST/ALT ratio of rats. Values are expressed as mean ± standard deviation (SD). Groups: Control, Normal saline; 5-FU, 5-FU 125 mg/kg; 5-FU + Pro, 5-FU 125 mg/kg + Propolis ethanolic extract 250 mg/kg/d; 5-FU + CLC, 5-FU 125 mg/kg + Colchicine 5 mg/kg/d. ^**^, ^***^, and ^****^ indicate p < 0.01, p < 0.001, and p < 0.0001, respectively.

The highest and lowest AST/ALT ratio were observed in the 5-FU and Control groups, respectively (Fig. 5). This ratio was significantly higher in the 5-FU and 5-FU + Pro groups than in the Control groups (p < 0.0001 and p < 0.01, respectively). This difference was also observed when comparing the 5-FU + Pro and 5-FU + CLC groups to the 5-FU group (p < 0.05 and p < 0.001, respectively). A significant difference was also observed between the treatment groups (Table 3).

#### Cardiac marker enzymes assay

##### Lactate dehydrogenase (LDH)

The highest and lowest LDH levels were observed in 5-FU + Pro and 5-FU + CLC groups, respectively. None of the groups had a significant difference (Table 3).

##### Creatinine kinase-MB (CK-MB)

The highest and lowest CK-MB levels were observed in 5-FU + Pro and 5-FU + CLC groups, respectively. None of the groups had a significant difference (Table 3).

### Biochemical analysis

#### Total anti-oxidant capacity (TAC) assay

**Figure 6:**
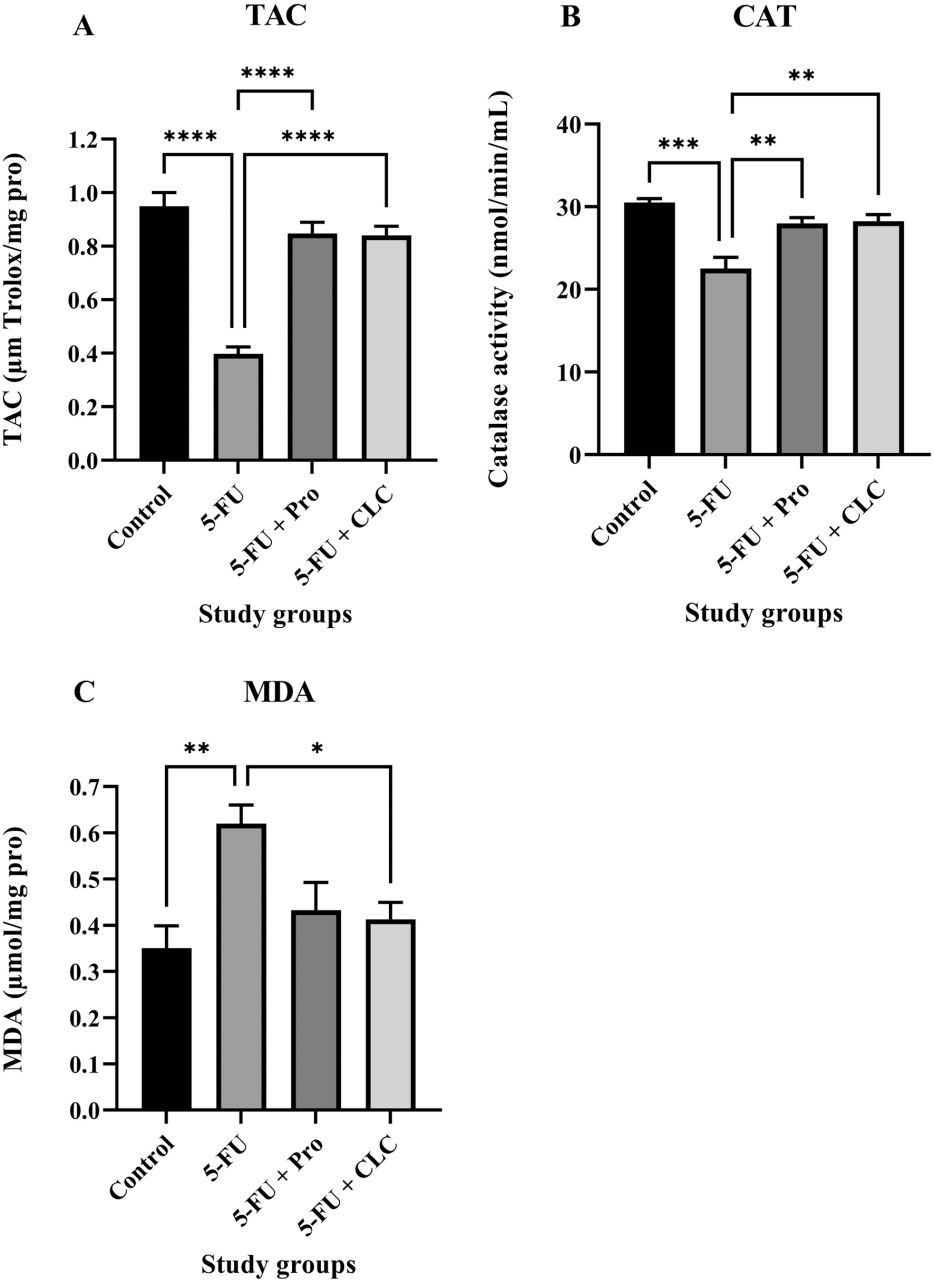
Effect of 5-flurouracil and propolis administration on the (**A**) TAC, (**B**) MDA, and (**C**) CAT status of the serum samples. Values are expressed as mean ± standard deviation (SD). Groups: Control, Normal saline; 5-FU, 5-FU 125 mg/kg; 5-FU + Pro, 5-FU 125 mg/kg + Propolis ethanolic extract 250 mg/kg/d; 5-FU + CLC, 5-FU 125 mg/kg + Colchicine 5 mg/kg/d. ^*^, ^**^, ^***^, and ^****^ indicate p < 0.05, p < 0.01, p < 0.001, and p < 0.0001, respectively.

In the TAC analysis, the highest and lowest levels of anti-oxidants were measured in the Control and 5-FU groups, respectively, with the Control group significantly higher than the 5-FU group (p < 0.0001). The TAC level in the 5-FU + CLC group was slightly lower than that of the 5-FU + Pro group. Also, the TAC level in the 5-FU + Pro and 5-FU + CLC groups was significantly higher than in the 5-FU group (p < 0.0001) (Fig. 6A).

#### Catalase (CAT) activity assay

In the CAT activity assay, the highest and lowest levels of CAT activity were measured in the Control and 5-FU groups, respectively, with the Control group significantly higher than the 5-FU group (p < 0.001). CAT activity was almost equal in the 5-FU + CLC and 5-FU + Pro groups, and it was significantly lower than in the 5-FU group (p < 0.01) (Fig. 6B).

#### Malondialdehyde (MDA) assay

In the MDA assay, the highest and lowest oxidant levels were measured in the 5-FU and Control groups, respectively, with the 5-FU group significantly higher than the Control group (p < 0.01). Also, the MDA level in the 5-FU + CLC group was significantly lower than 5-FU (p < 0.05). Moreover, the MDA level in the 5-FU + Pro group was slightly higher than in the 5-FU + CLC group (Fig. 6C).

### The histopathological changes in heart tissue

#### Necrosis

**Figure 7:**
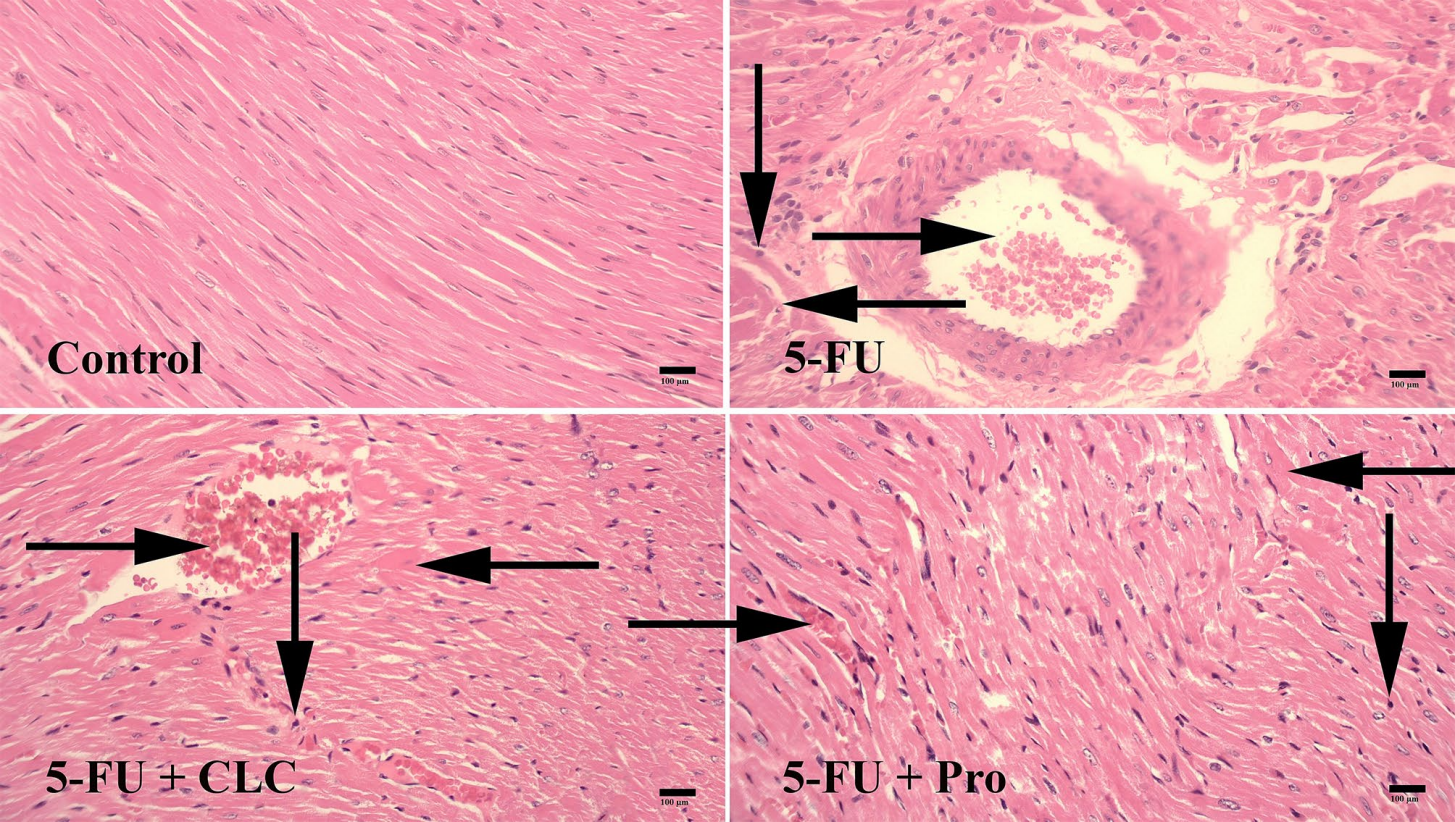
Effect of 5-fluorouracil and propolis administration on the histopathological changes of heart tissue samples. Groups: Control, Normal saline; 5-FU, 5-FU 125 mg/kg; 5-FU + Pro, 5-FU 125 mg/kg + Propolis ethanolic extract 250 mg/kg/d; 5-FU + CLC, 5-FU 125 mg/kg + Colchicine 5 mg/kg/d. Normal conditions in the Control group. Note hyperemia (rightward arrows), necrosis (downward arrows), and hyalinization (leftward arrows) in the 5-FU, 5-FU + Pro, and 5-FU + CLC groups. Hematoxylin and Eosin (H&E) staining. ×100 magnification.

In the study performed on heart tissue samples, the highest and lowest necrosis were observed in 5-FU and Control groups, respectively. The 5-FU + CLC, 5-FU + Pro, and 5-FU groups were significantly more necrotic than the Control group (p < 0.01, p < 0 0.01, and p < 0.001, respectively) (Table 4) (Fig. 7).

**Table 4:** Histopathological parameters of study groups.

Study groups	Histopathological parameters		
	Necrosis	Hyperemia	Hyalinization
Control	9.65 ± 0.32	8.00 ± 0.0	10.80 ± 0.32
5-FU	29.20 ± 0.85***	29.75 ± 0.82****	25.70 ± 0.67**
5-FU + Pro	22.40 ± 0.57**	16.50 ± 0.68**^, ##, $$^	22.75 ± 0.63**
5-FU + CLC	20.75 ± 0.63**	27.75 ± 0.92****^, $$^	22.75 ± 0.63**

Values are expressed as mean ± standard deviation (SD). Groups: Control, Normal saline; 5-FU, 5-FU 125 mg/kg; 5-FU + Pro, 5-FU 125 mg/kg + Propolis ethanolic extract 250 mg/kg/d; 5-FU + CLC, 5-FU 125 mg/kg + Colchicine 5 mg/kg/d. **, ***, and **** Indicate statistically significant difference compared to the control group (p < 0.01, p < 0.001, and p < 0.0001, respectively). ^##^ Indicate statistically significant difference compared to the 5-FU group (p < 0.01). ^$$^ Indicate statistically significant difference between the treatment groups (p < 0.01).

#### Hyperemia

The highest and lowest rates of hyperemia were observed in the 5-FU and Control groups, respectively. The 5-FU + Pro, 5-FU + CLC, and 5-FU groups were significantly more hyperemic than the Control group (p < 0.01, p < 0 0.0001, and p < 0.0001, respectively). Also, the hyperemia in the 5-FU and 5-FU + CLC groups were significantly higher than in the 5-FU + Pro group (p < 0.01) (Table 4) (Fig. 7).

#### Hyalinization

The highest and lowest hyalinization were observed in the 5-FU and Control groups, respectively. The 5-FU + CLC, 5-FU + Pro, and 5-FU groups were significantly more hyalinized than the Control group (p < 0.01) (Table 4) (Fig. 7).

### Gene expression

#### TNF-α expression

**Figure 8:**
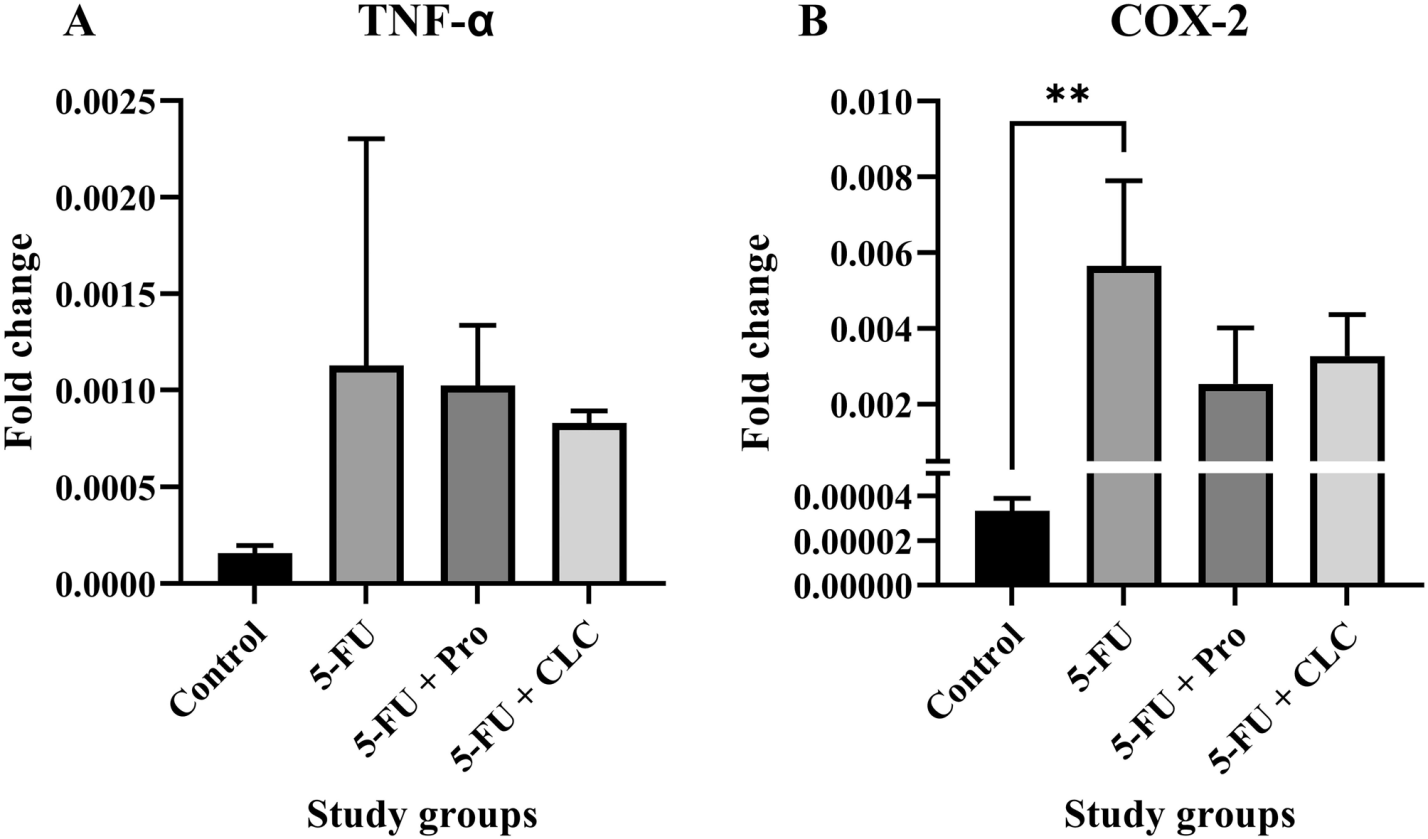
Effect of 5-fluorouracil and propolis administration on the expression of (**A**) *TNF-α* and (**B**) *COX-2*. Values are expressed as mean ± standard deviation (SD). Groups: Control, Normal saline; 5-FU, 5-FU 125 mg/kg; 5-FU + Pro, 5-FU 125 mg/kg + Propolis ethanolic extract 250 mg/kg/d; 5-FU + CLC, 5-FU 125 mg/kg + Colchicine 5 mg/kg/d. ^**^ indicate p < 0.01.

The lowest and highest levels of TNF-α expression were observed in the Control and 5-FU groups, respectively. *TNF-α* expression was not significantly different in any group. Also, *TNF-α* expression in the 5-FU + CLC group was higher than in the 5-FU + Pro group (Fig. 8A).

#### COX-2 expression

The highest *COX-2* expression was observed in the 5-FU group and the lowest in the Control group. Its expression was significantly lower in the Control group than in the 5-FU group (p < 0.01). *COX-2* expression is higher in the 5-FU + CLC group than in the 5-FU + Pro group (Fig. 8B).

## Discussion

Propolis is a resin produced by bees from various plants with different properties, such as anticancer, anti-inflammatory, antibacterial, and anti-oxidant, with no known severe side effects. This natural agent mainly exerts its effects by inhibiting mitochondrial stress, cell proliferation, and growth, stimulating cell cycle arrest, and inducing apoptosis^43^.

The MTT results showed that propolis was beneficial for rats treated with 5-FU in a dose-dependent manner. Nevertheless, as the propolis dose increased, its positive effect was diminished due to a synergistic toxic effect between the 5-FU and propolis itself. In a study, the cytotoxic effect of isorhamnetin on various gastric cancer cells was investigated. The study results showed that, depending on the time and dose of treatment, isorhamnetin could inhibit two multidrug-resistant gastric cancer cell lines and significantly increase the susceptibility of gastric tumor cells to chemotherapy^44^. Another study found that concomitant use of apigenin and 5-FU could decrease the viability of colorectal cancer cells^45^.

In this study, we examined the relative weight of rats’ heart tissues. The results showed that the highest weight was observed in the 5-FU + Pro group, and the lowest was observed in the 5-FU group. The results showed that the decrease in the relative heart weight due to the use of 5-FU could be significantly compensated by propolis administration. However, CLC did not significantly differ in relative heart weight compared to the Control group. One study concluded that using silymarin, an anticancer flavonoid, prevented total body weight loss by reducing the peroxidative activity of doxorubicin^46^. The results of other studies also demonstrated that 5-FU administration would reduce’ bodyweight, which could be due to damage to the liver^47^ or intestines tissues^48^.

In the ECG monitoring of study samples, it was deduced that using 5-FU significantly altered the ECG components compared to the Control group. These changes include QRS interval prolongation, ST-segment elevation, and QTc increment. The use of propolis could bring these changes back to normal levels. The use of CLC also had a positive effect and returned these changes to normal levels. In a study, Aygun et al.^49^ showed that doxorubicin reduced P and QRS wavelengths, prolonged QT interval, and elevated ST segment. A study on fluoropyrimidines found that they exert a toxic effect on the heart muscle, depending on the dose and type of drug. For example, 5-FU at a lower dose had a more toxic effect on the myocardium than capecitabine^13^.

In our study, the number of blood cells and hemoglobin in the 5-FU group was lower than in other groups. The concomitant use of propolis largely compensated for this discrepancy. CLC is also effective in improving blood cell counts and hemoglobin levels to a large extent, similar to that of propolis. It has previously been observed that propolis can significantly increase the number of blood cells^50^. In a study of metrifonate toxicity, it was found that blood cell counts were greatly reduced, and concomitant use of propolis improved and primarily compensated for the negative changes in hematologic parameters^51^.

Cardiac and serological biomarkers were also examined, and the results showed a significant increase in AST/ALT ratio and LDH and CKMB levels in the 5-FU group. Although concomitant administration of propolis could scarcely alleviate this negative impact, the use of CLC returned the level of these biomarkers nearly to normal values. A study showed that propolis containing chrysin could reduce and normalize the cardiac biomarkers increment induced by methotrexate^52^. Furthermore, elevated cardiac and serological biomarkers in the doxorubicin-treated rats were greatly reduced when propolis was simultaneously administered^24^. A study on reducing the toxicity of doxorubicin with pinocembrin, a flavonoid containing propolis, showed similar results, normalizing the cardiac biomarkers^53^. Regarding the AST/ALT ratio, which was almost doubled in our study in the 5-FU group compared to the Control group, there is growing evidence supporting this hypothesis that AST/ALT ratio would increase in cardiac events, especially ST-elevation MI (STEMI)^54,55^.

Catalase, an anti-oxidant enzyme, is a catalyst for the breakdown of H_2_O_2_ into water and oxygen, preventing oxidative stress-induced cell damage^56^. MDA is a measurable biomarker obtained by decomposing unsaturated fatty acids into free radicals^38^. This study observed that the increase in MDA level in the 5-FU group could be significantly counterbalanced and reduced by administering Propolis and CLC. Moreover, a decrease in the CAT and TAC levels was observed in the 5-FU group compared to the Control group, counterbalanced when propolis and CLC were administered to the treatment groups. In a study, the use of doxorubicin increased MDA production and peroxidative damage in treated rats, while concomitant use of propolis reduced MDA and peroxidative damage to rat mitochondria^57^. In another study on the effect of propolis in reducing oxidative stress induced by gentamicin, it was observed that the levels of hepatic and renal oxidative stress markers, such as CAT, and MDA, were significantly decreased and increased, respectively, when propolis was applied to the study samples^58^.

Histological alterations of the heart tissue samples were studied for necrosis, hyperemia, and hyalinization. The highest incidence of necrosis, hyperemia, and hyalinization was observed in the 5-FU group. However, simultaneous use of propolis or CLC could not significantly improve this destruction. A previous study found that 5-FU-induced cardiac toxicity was widespread in study animals and included multifocal myofiber necroses, vascular and valve changes, multiple myocardial interstitial hemorrhages, and pericarditis, especially in the left ventricle, and inflammatory responses^59^. In histological studies performed by Gelen et al.^60^, necrosis and hyperemia in the kidney and vascular tissue of the group treated with 5-FU were immensely presented. On the other hand, concomitant use of hesperidin or curcumin (more effective than hesperidin) with 5-FU in other groups reduced the extent of these tissue changes^60^.

In the molecular analysis performed in this study, the expression of *TNF-α* and *COX-2* in the 5-FU group was increased compared to the Control group. With the use of propolis or CLC, the expression of *COX-2* was significantly reduced, but yet higher than the Control group. Nonetheless, administration of propolis or CLC had little effect on *TNF-α* expression, slightly decreasing it. A study on the effect of capecitabine in the treatment of gastric cancer showed that this medication could increase the expression of the *COX-2*, which is greatly reduced by the concomitant use of isorhamnetin^44^. A systematic review demonstrated that following propolis use, serum levels of TNF-α and CRP were significantly reduced^61^. Moreover, consumption of 5-FU significantly upregulated COX-2 expression, but simultaneous treatment with propolis counterbalanced and downregulated its expression in a dose-dependent manner^43^.

## Conclusion

Although the findings of this study should be validated in future preclinical and clinical studies, our results implied that the administration of propolis ethanolic extract could significantly ameliorate the destructive and cardiotoxic effects of chemotherapeutic medications including 5-fluorouracil. Moreover, this beneficial effect was observed to be more potent than colchicine, which is an approved cardioprotective drug.

## Data Availability

The datasets used and/or analyzed during the current study are available from the corresponding author on reasonable request.
