# Author Correction: The effect of propolis on 5-fluorouracil-induced cardiac toxicity in rats

**DOI:** 10.1038/s41598-022-17003-7

**Published:** 2022-07-22

**Authors:** Mohammad Barary, Rezvan Hosseinzadeh, Sohrab Kazemi, Jackson J. Liang, Razieh Mansoori, Terence T. Sio, Mohammad Hosseini, Ali Akbar Moghadamnia

**Affiliations:** 1 Student Research Committee, Virtual School of Medical Education and Management, Shahid Beheshti University of Medical Sciences, Tehran, Iran; 2 Students’ Scientific Research Center (SSRC), Tehran University of Medical Sciences, Tehran, Iran; 3 Student Research Committee, Babol University of Medical Sciences, Babol, Iran; 4 Cellular and Molecular Biology Research Center, Health Research Center, Babol University of Medical Sciences, Babol, Iran; 5 Division of Cardiovascular Medicine, Cardiac Arrhythmia Service, University of Michigan, Ann Arbor, MI USA; 6 Department of Radiation Oncology, Mayo Clinic, Phoenix, AZ USA; 7 Department of Veterinary Parasitology, Babol-Branch, Islamic Azad University, Babol, Iran

Correction to: *Scientific Reports* https://doi.org/10.1038/s41598-022-12735-y, published online 23 May 2022

The original version of this Article contained an error in the spelling of the author Razieh Mansoori which was incorrectly given as Razieh Mansouri.

The original Article has been corrected.

